# Complete genome sequence of *Methanoplanus petrolearius* type strain (SEBR 4847^T^)

**DOI:** 10.4056/sigs.1183143

**Published:** 2010-10-27

**Authors:** Evelyne Brambilla, Olivier Duplex Ngatchou Djao, Hajnalka Daligault, Alla Lapidus, Susan Lucas, Nancy Hammon, Matt Nolan, Hope Tice, Jan-Fang Cheng, Cliff Han, Roxanne Tapia, Lynne Goodwin, Sam Pitluck, Konstantinos Liolios, Natalia Ivanova, Konstantinos Mavromatis, Natalia Mikhailova, Amrita Pati, Amy Chen, Krishna Palaniappan, Miriam Land, Loren Hauser, Yun-Juan Chang, Cynthia D. Jeffries, Manfred Rohde, Stefan Spring, Johannes Sikorski, Markus Göker, Tanja Woyke, James Bristow, Jonathan A. Eisen, Victor Markowitz, Philip Hugenholtz, Nikos C. Kyrpides, Hans-Peter Klenk

**Affiliations:** 1 DSMZ - German Collection of Microorganisms and Cell Cultures GmbH, Braunschweig, Germany; 2 HZI – Helmholtz Centre for Infection Research, Braunschweig, Germany; 3 Los Alamos National Laboratory, Bioscience Division, Los Alamos, New Mexico, USA; 4 DOE Joint Genome Institute, Walnut Creek, California, USA; 5 Biological Data Management and Technology Center, Lawrence Berkeley National Laboratory, Berkeley, California, USA; 6 Oak Ridge National Laboratory, Oak Ridge, Tennessee, USA; 7 University of California Davis Genome Center, Davis, California, USA

**Keywords:** obligately anaerobic, mesophilic, hydrogen, methane, Gram-negative, *Methanomicrobiaceae*, *Euryarchaeota*, GEBA

## Abstract

*Methanoplanus petrolearius* Ollivier *et al.* 1998 is the type strain of the genus *Methanoplanus*. The strain was originally isolated from an offshore oil field from the Gulf of Guinea. Members of the genus *Methanoplanus* are of interest because they play an important role in the carbon cycle and also because of their significant contribution to the global warming by methane emission in the atmosphere. Like other archaea of the family *Methanomicrobiales*, the members of the genus *Methanoplanus* are able to use CO_2_ and H_2_ as a source of carbon and energy; acetate is required for growth and probably also serves as carbon source. Here we describe the features of this organism, together with the complete genome sequence and annotation. This is the first complete genome sequence of a member of the family *Methanomicrobiaceae* and the sixth complete genome sequence from the order *Methanomicrobiales*. The 2,843,290 bp long genome with its 2,824 protein-coding and 57 RNA genes is a part of the *** G****enomic* *** E****ncyclopedia of* *** B****acteria and* *** A****rchaea * project.

## Introduction

Strain SEBR 4847^T^ (= DSM 11571 = OCM 486) is the type strain of *Methanoplanus petrolearius* [1]. This strain was isolated from an offshore oil-producing well in the Gulf of Guinea, Africa [1]. Currently, the genus *Methanoplanus* contains three species: *M. petrolearius*, the type species *M. limicola* (isolated from an Italian swamp containing drilling waste near Baia in the Naples Area), and *M. endosymbiosus* (isolated from the marine ciliate *Metopus contortus*) [1]. The genus name derived from the Latin word “*methanum*”, and the adjective “*planus*”, meaning a flat plate, which refers to its flat cell morphology [1,2]. *Methanoplanus* therefore means “methane (-producing) plate”. The species epithet *petrolearius* derives from the Latin word “*petra*”, rock and the adjective “*olearius*”, which relates to vegetable oil [1]. “*Petrolearius*” means therefore related to mineral oil, referring to its origin of isolation [1]. No additional cultivated strains belonging to the species *M. petrolearius* have been described thus far. *M*. *petrolearius* SEBR 4847^T^ is like other methanogens, strictly anaerobic. Here we present a summary classification and a set of features for *M*. *petrolearius* strain SEBR 4847^T^, together with the description of the complete genomic sequencing and annotation.

## Classification and features

The type strains of the two other species in the genus *Methanoplanus* share an average of 93.5% 16S rRNA gene sequence identity with strain SEBR 4847^T^ [1,2]. The 16S rRNA gene sequence of the strain SEBR 4847^T^ shows 99% identity with an uncultured environmental 16S rRNA gene sequence of the clone KO-Eth-A (AB236050) obtained from the marine sediment [3]. The 16S rRNA gene sequences similarities of the strain SEBR 4847^T^ to metagenomic libraries (env_nt) were all 83% or less, (status August 2010), indicating that members of the species, genus and even family are poorly represented in the habitats screened thus far.

**Figure 1:**
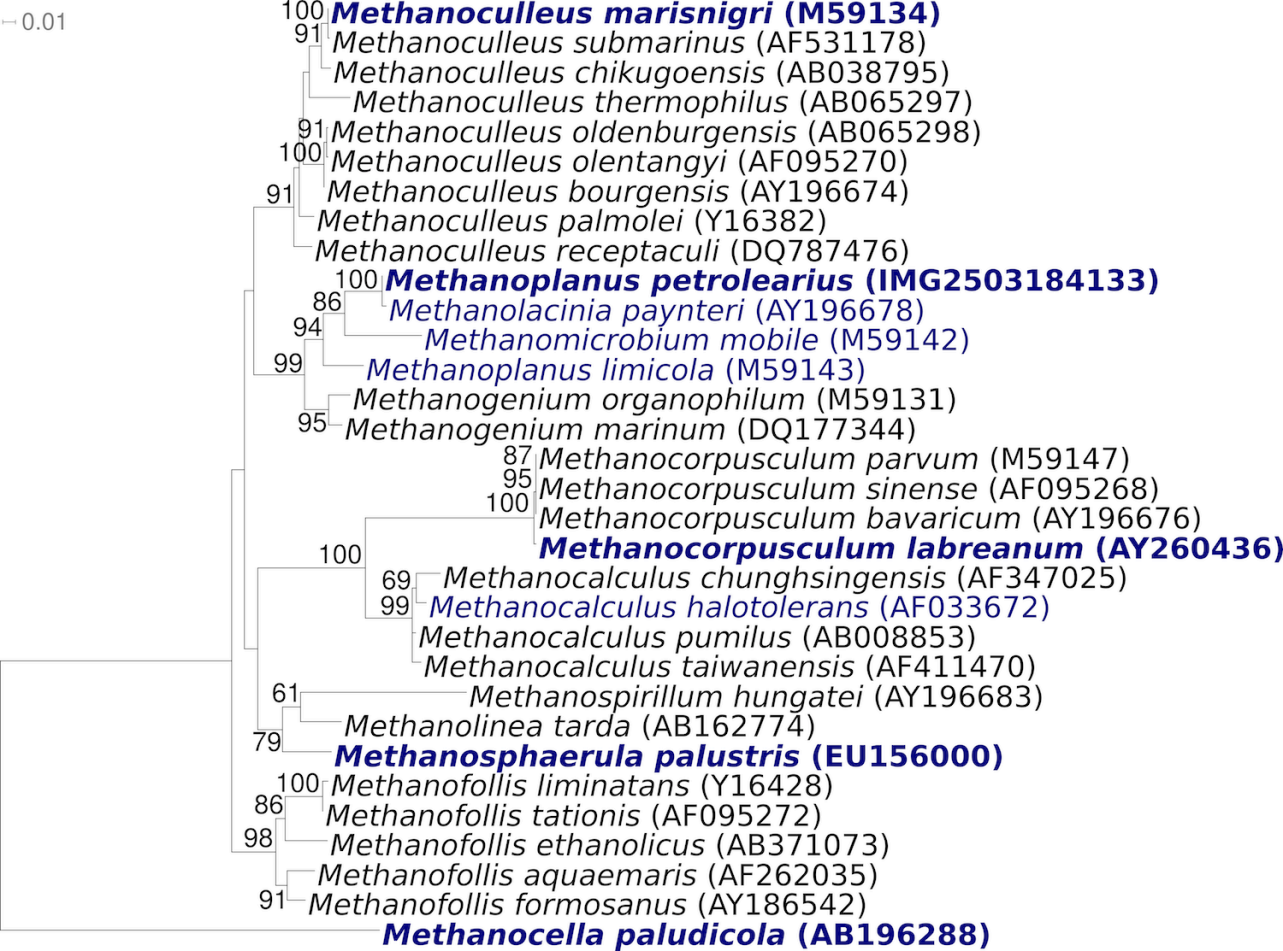
Phylogenetic tree highlighting the position of *M. petrolearius* SEBR 4847^T^ relative to the other type strains within the order *Methanomicrobiales*. The tree was inferred from 1,275 aligned characters [4,5] of the 16S rRNA gene sequence under the maximum likelihood criterion [6] and rooted with *Methanocellales* [7]. The branches are scaled in terms of the expected number of substitutions per site. Numbers above branches are support values from 350 bootstrap replicates [8] if larger than 60%. Lineages with type strain genome sequencing projects registered in GOLD [9] are shown in blue, published genomes in bold [10,11] and GenBank accessions CP001338 (for *Methanosphaera palustris* E1-9c) and AP011532 (for *Methanocella paludicola*).

Figure 1 shows the phylogenetic neighborhood of *M. petrolearius* SEBR 4847^T^ in a 16S rRNA based tree. The sequences of the two identical 16S rRNA gene copies in the genome do not differ from the previously published 16S rRNA sequence generated from DSM 11571 (U76631), which contained four ambiguous base calls.

**Figure 2:**
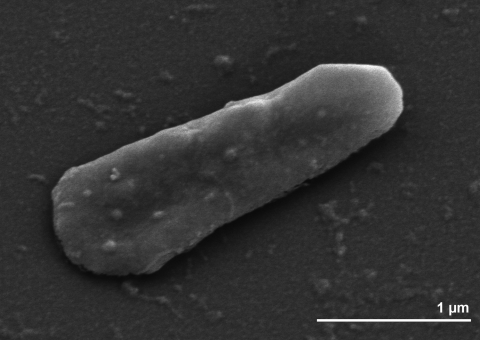
Scanning electron micrograph of *M. petrolearius* SEBR 4847^T^

The cells of strain SEBR 4847^T^ stain Gram-negative, but archaea do not have a Gram-negative type of cell wall with an outer envelope. Cells occur singly or in pairs and are irregularly disc-shaped of 1 to 3 µm size (Figure 2 and Table 1). A similar shape was found for two other strains of the genus *Methanoplanus* [1,2,24]. Strain SEBR 4847^T^ was originally described as non-motile [1], however, in samples of this strain kept in the DSMZ culture collection motile cells were frequently detected in young cultures (H. Hippe, personal communication). The genome sequence of SEBR 4847^T^ contains numerous genes encoding flagellins (Mpet_2052 - Mpet2054, Mpet_2057) and chemotaxis proteins (Mpet_2064 – Mpet_2069), which is in line with the observation of motility in this species. Round colonies of 1-2 mm are observed after three weeks of incubation on solid agar medium. The generation time of strain SEBR 4847^T^ is about 10 hours under optimal conditions [1]. Strain SEBR 4847^T^ grows optimally at 37°C, the temperature range for growth being 28-43°C. No growth was observed at 25°C or 45°C [1]. The optimum pH is 7.0; growth occurs from pH 5.3 to 8.4. The optimum NaCl concentration for growth is between 1 and 3% NaCl with growth occurring at NaCl concentrations ranging from 0 to 5% [1]. Substrates for growth of strain SEBR 4847^T^ are H_2_ + CO_2_, formate and CO_2_ + 2-propanol [1]. Strain SEBR 4847^T^ does not utilize methanol, trimethylamine, lactate, glucose, CO_2_ + 1-propanol, CO_2_ + 1-butanol and isobutyrate [1]. Acetate is required for growth as carbon source and yeast extract is stimulatory [1]. Addition of acetate reduces the lag time [25]. The addition of acetate slightly increases the amount of H_2_ available, theoretically [26,27]. When H_2_ is limiting and sulfate is in excess, sulfate reducers compete with methanogens and homoacetogens for the available H_2_ [27]. The sulfate reducers can out-compete hydrogenotrophic methanogens, due to a higher affinity [28] and higher activity of hydrogenase and the energetically more favorable reduction of sulfate [29]. Similar features were observed for *M. limicola* and *M. endosymbiosus* [1,2,24].

**Table 1:** Classification and general features of *M. petrolearius* SEBR 4847^T^ according to the MIGS recommendations [12]

**MIGS ID**	**Property**	**Term**	**Evidence code**
	Current classification	Domain *Archaea*	TAS [13]
		Phylum *Euryarchaeota*	TAS [14,15]
		Class *Methanomicrobia*	TAS [16]
		Order *Methanomicrobiales*	TAS [17-19]
		Family *Methanomicrobiaceae*	TAS [17,18]
		Genus *Methanoplanus*	TAS [2,20]
		Species *Methanoplanus petrolearius*	TAS [1,21]
		Type strain SEBR 4847	TAS [1]
	Gram stain	negative	TAS [2]
	Cell shape	disc-shaped, irregular single or in pairs	TAS [1]
	Motility	motile	IDA
	Sporulation	not reported	NAS
	Temperature range	28-43°C	TAS [1]
	Optimum temperature	37°C	TAS [1]
	Salinity	1-3% NaCl	TAS [1]
MIGS-22	Oxygen requirement	anaerobic obligate	TAS [1]
	Carbon source	acetate, CO_2_, formate	TAS [1]
	Energy source	H_2_ + CO_2_, formate and CO_2_ + 2-propanol	TAS [1]
MIGS-6	Habitat	offshore oil field	TAS [1]
MIGS-15	Biotic relationship	not reported	NAS
MIGS-14	Pathogenicity	not reported	NAS
	Biosafety level	1	TAS [22]
	Isolation	subsurface ecosystem	TAS [1]
MIGS-4	Geographic location	offshore oil field, Gulf of Guinea, West Africa	TAS [1]
MIGS-5	Sample collection time	1997 or before	TAS [1]
MIGS-4.1 MIGS-4.2	Latitude Longitude	not reported	NAS
MIGS-4.3	Depth	not reported	NAS
MIGS-4.4	Altitude	not reported	NAS

Evidence codes - IDA: Inferred from Direct Assay (first time in publication); TAS: Traceable Author Statement (i.e., a direct report exists in the literature); NAS: Non-traceable Author Statement (i.e., not directly observed for the living, isolated sample, but based on a generally accepted property for the species, or anecdotal evidence). These evidence codes are from of the Gene Ontology project [23]. If the evidence code is IDA, then the property was directly observed by one of the authors or an expert mentioned in the acknowledgements

### Chemotaxonomy

At the time of writing, no reports have been published describing the composition of the cell envelope of the strain SEBR 4847^T^. However, for the two other species in the genus *Methanoplanus*, *M. limicola* and *M. endosymbiosus*, several chemotaxonomic features have been reported [2,24]. Preparations of the cell envelope from *M. limicola* and *M. endosymbiosius* revealed the presence of a dominant band that appeared to be a glycoprotein when cells were disrupted in 2% SDS [2,24]. *Methanoplanus* spp. possesses a mixture of C_20_C_20_ and C_40_C_40_ core ethers [30]. For comparison, similar mixtures were also detected in other members of the family *Methanomicrobiaceae*: *Methanogenium cariaci,* *Methanogenium marisnigri* and *Methanogenium thermophilicum*, while C_20_C_25_ was absent in these species [30].

## Genome sequencing and annotation

### Genome project history

This organism was selected for sequencing on the basis of its phylogenetic position [31], and is part of the *** G****enomic* *** E****ncyclopedia of* *** B****acteria and* *** A****rchaea * project [32]. The genome project is deposited in the Genome OnLine Database [9] and the complete genome sequence is deposited in GenBank. Sequencing, finishing and annotation were performed by the DOE Joint Genome Institute (JGI). A summary of the project information is shown in Table 2.

**Table 2:** Genome sequencing project information

**MIGS ID**	**Property**	**Term**
MIGS-31	Finishing quality	Finished
MIGS-28	Libraries used	Tree genomic libraries: 454 pyrosequence standard library, paired end 454 library (9.5 kb insert size), Illumina GAii shotgun library
MIGS-29	Sequencing platforms	454 GS FLX Titanium, Illumina GAii
MIGS-31.2	Sequencing coverage	67.9 × pyrosequence, 52.2 × Illumina
MIGS-30	Assemblers	Newbler version 2.3-PreRelease-09-14-2009, Velvet, phrap
MIGS-32	Gene calling method	Prodigal 1.4, GenePRIMP
	INSDC ID	CP002117
	Genbank Date of Release	September 17, 2010
	NCBI project ID	40773
	GOLD ID	Gc01372
	Database: IMG-GEBA	2503128011
MIGS-13	Source material identifier	DSM 11571
	Project relevance	Tree of Life, GEBA

### Growth conditions and DNA isolation

*M. petrolearius* SEBR 4847^T^, DSM 11571, was grown anaerobically in DSMZ medium 141 (Methanogenium medium) [33] at 37°C. DNA was isolated from 0.2 g of cell paste using a phenol/chloroform extraction after cell lysis with a mixture of lysozyme and mutanolysin.

### Genome sequencing and assembly

The genome was sequenced using a combination of Illumina and 454 sequencing platforms. All general aspects of library construction and sequencing can be found at the JGI website (http://www.jgi.doe.gov/). Pyrosequencing reads were assembled using the Newbler assembler Version 2.3 Pre-Release-09-14-2009 (Roche). The initial Newbler assembly consisted of 21 contigs in one scaffold that was converted into a phrap assembly by making fake reads from the consensus sequence. Illumina GAii sequencing data (148.5Mb) was assembled with Velvet [34] and the consensus sequences were shredded into 1.5 kb overlapped fake reads and assembled together with the 454 data. The draft assembly was based on 173.4 Mb of 454 data and all of the 454 paired end data. Newbler parameters are -consed -a 50 -l 350 -g -m -ml 20. The Phred/Phrap/Consed software package (www.phrap.com) was used for sequence assembly and quality assessment of the genome sequence. After the shotgun stage, reads were assembled with parallel phrap (High Performance Software, LLC). Possible mis-assemblies were corrected with gapResolution (http://www.jgi.doe.gov/), Dupfinisher, or sequencing cloned bridging PCR fragments with subcloning or transposon bombing (Epicentre Biotechnologies, Madison, WI) [35]. Gaps between contigs were closed by editing in Consed, by PCR and by Bubble PCR primer walks (J.-F.Chang, unpublished). A total of 139 additional reactions were necessary to close gaps and to raise the quality of the finished sequence. Illumina reads were also used to correct potential base errors and increase consensus quality using a software Polisher developed at JGI [36]. The error rate of the completed genome sequence is less than 1 in 100,000. Together, the combination of the Illumina and 454 sequencing platforms provided 120.1× coverage of the genome. The final assembly of the genoe contains 590,575 pyrosequences and 4,125,153 Illumina reads.

### Genome annotation

Genes were identified using Prodigal [37] as part of the Oak Ridge National Laboratory genome annotation pipeline, followed by a round of manual curation using the JGI GenePRIMP pipeline [38]. The predicted CDSs were translated and used to search the National Center for Biotechnology Information (NCBI) nonredundant database, UniProt, TIGRFam, Pfam, PRIAM, KEGG, COG, and InterPro databases. Additional gene prediction analysis and functional annotation was performed within the Integrated Microbial Genomes - Expert Review (IMG-ER) platform [39].

## Genome properties

**Figure 3:**
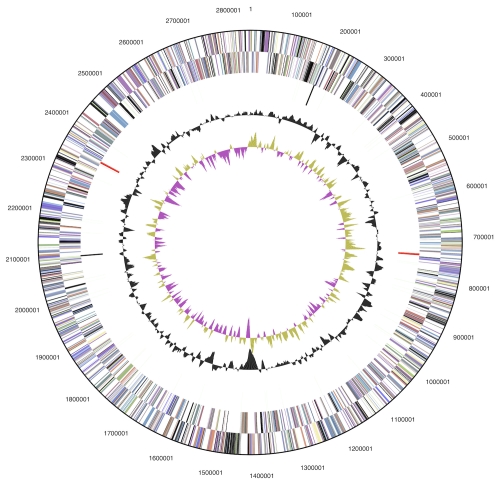
Graphical circular map of the genome. From outside to the center: Genes on forward strand (color by COG categories), Genes on reverse strand (color by COG categories), RNA genes (tRNAs green, rRNAs red, other RNAs black), GC content, GC skew.

The genome consists of a 2,843,290 bp long chromosome with a 47.4% GC content (Table 3 and Figure 3). Of the 2,881 genes predicted, 2,825 were protein-coding genes, and 57 RNAs; thirty nine pseudogenes were also identified. The majority of the protein-coding genes (61.2%) were assigned a putative function while the remaining ones were annotated as hypothetical proteins. The distribution of genes into COGs functional categories is presented in Table 4.

**Table 3:** Genome Statistics

**Attribute**	**Value**	**% of Total**
Genome size (bp)	2,843,290	100.00%
DNA coding region (bp)	2,501,893	87.99%
DNA G+C content (bp)	1,347,696	47.40%
Number of replicons	1	
Extrachromosomal elements	0	
Total genes	2,881	100.00%
RNA genes	57	1.98%
rRNA operons	2	
Protein-coding genes	2,824	98.02%
Pseudo genes	39	1.35%
Genes with function prediction	1,793	62.24%
Genes in paralog clusters	550	19.10%
Genes assigned to COGs	1,939	67.30%
Genes assigned Pfam domains	2,000	69.42%
Genes with signal peptides	492	17.10%
Genes with transmembrane helices	886	30.75%
CRISPR repeats	0	

**Table 4:** Number of genes associated with the general COG functional categories

**Code**	**value**	**%age**	**Description**
J	150	7.1	Translation, ribosomal structure and biogenesis
A	0	0.0	RNA processing and modification
K	106	5.0	Transcription
L	80	3.8	Replication, recombination and repair
B	2	0.1	Chromatin structure and dynamics
D	18	0.9	Cell cycle control, cell division, chromosome partitioning
Y	0	0.0	Nuclear structure
V	28	1.3	Defense mechanisms
T	136	6.5	Signal transduction mechanisms
M	67	3.2	Cell wall/membrane/envelope biogenesis
N	54	2.6	Cell motility
Z	1	0.0	Cytoskeleton
W	0	0.0	Extracellular structures
U	32	1.5	Intracellular trafficking and secretion, and vesicular transport
O	80	3.8	Posttranslational modification, protein turnover, chaperones
C	185	8.8	Energy production and conversion
G	70	3.3	Carbohydrate transport and metabolism
E	155	7.4	Amino acid transport and metabolism
F	61	2.9	Nucleotide transport and metabolism
H	162	7.7	Coenzyme transport and metabolism
I	22	1.1	Lipid transport and metabolism
P	143	6.8	Inorganic ion transport and metabolism
Q	7	0.3	Secondary metabolites biosynthesis, transport and catabolism
R	278	13.2	General function prediction only
S	267	12.7	Function unknown
-	942	32.7	Not in COGs
